# The Operative Treatment of Pleuritic Exudations

**Published:** 1876-05

**Authors:** 


### MISCELLANEOUS.

---

### The Operative Treatment of Pleuritic Exudations.

---

"An important contribution on this subject appears in the the recent volume of the 'Berlin Charite Reports' (*Charite Annalen*, edited by Dr. Mehlhausen; Hirschwald's, Berlin, 1876, pp. 752). It is from the pen of Dr. Ewald, and is founded on the observation of 250 cases which were admitted into the medical clinical wards of Professor Frerichs during a period of fifteen years. from June 1, 1860 to June 1, 1875. This question is raised less with a view to the recording of individual cases of serous effusion which have been successfully aspirated, or of purulent cases which have been successfully and freely opened, but rather in the view of laying before the profession the results of a large number of cases treated by the same individual under similar circumstances and surroundings, and during a given period of years. Unfortunately, such statistics are comparatively rare, though a considerable number of single cases are scattered throughout the extensive literature bearing on this subject. Dr. Ewald sets out with the assumption 'that there are cases in which the timely and artificial evacuation of the collected fluid alone can save from an either directly or indirectly unfavorable termination.'

It is in the hope of contributing some reliable data as to the 'when' and the 'how' this interference shall be most aptly exercised that the author has undertaken a publication, which we shall now proceed to consider. Of the 250 cases 204 were cases of serous effusion. We will take these first. It appears that during the years 1860–70 no cases of serous effusion were tapped, but were treated on general principles. The figures do not include some complicated cases in which the effusion was due either to tuberculosis or cancer, such cases being quite outside the question of surgical treatment. He deducts, also, some of the slighter cases, about which there never was any question of mechanical interference; and in this way the numbers are reduced to 143 cases with four deaths, during a period of fifteen years. As before said, serous effusions were not tapped previous to the year 1870, but between this date and 1875 twenty-six of the cases were tapped, all recovering. This small proportion of operated cases will at once show that only in certain cases was interference considered desirable; and, indeed, the following are his two chief indications: First, danger to life from pressure on vital organs; secondly, the apparent uselessness of drugs, if, after a three weeks' trial, they have failed to carry off the effusion. Concerning the first indication authors are fairly unanimous, but less so with regard to the second, the chief differences of opinion being as to the re-collection of fluid after tapping, its tendency to become purulent, and the actual capability of the lung to expand. As regards the re-collection of fluid after tapping, this our author considers as dependent on the time at which the tapping is performed; if it be done while the pleura has a natural tendency to exudation, tapping can in no way modify it—may possibly even increase it; for, as Wintrich says, tapping only draws off a product of disease, and not the disease itself. The indication, therefore, is to wait until physical examination determines the height of the disease, as judged by a standstill of the previously increasing dullness, which generally takes place in the third or fourth week. The tendency to become purulent is generally ascribed to two chief causes—first, directly to the operation; and, secondly, to the determining influence of the draining away of the fluid. Dr. Ewald, however, takes another view. Of thirty-five cases nine became purulent, but of these four times the fluid was turbid from the first, in one a canula was purposely left in the chest, and in the remaining four the operation was done much earlier than he now advises. The other cases, twenty-six in number, were tapped after the fourth week, and all continued serous throughout. In face of these facts, he considers himself justified in formulating that tapping does not tend to the conversion of a serous into a purulent pleurisy, provided due care be exercised in the time and mode of operating, the proper disinfection of the instruments used, and in carefully excluding air from the chest.

There can be no doubt as Trousseau has shown, that certain exudations tend of themselves to become purulent in time. It is especially the case in secondary pleurisies; and so, if from necessity the fluid has been drawn off early, while still serous, its becoming purulent at a latter stage must be ascribed rather to a natural pathological process than to the operation of thoracentesis. Next comes the question, After how long will a compressed lung re-expand? Authors vary on this point, and some exceptional cases, no doubt, show that even after a six month's compression the lung may unfold and regain its normal proportions. As a rule, however, one may state eight weeks as the average time; the date of the first tapping and the amount of the evacuated fluid being factors of no small moment in the estimation of this point. Dr. Ewald lays considerable stress on the mode of operating; he disapproves of the so-called suction instruments, as uncertain and unequal in action. As a rule, it is best to trust to the pressure from within, and only in some rare cases, when the fluid is viscid or thick, to apply a gentle suction. Deep inspirations, by expanding the lung, will generally force out as much of the fluid as it is wise or proper to withdraw; and it not unfrequently happens, should any be left behind, that it is shortly reabsorbed. He has only had one case in which the albuminous expectoration prescribed by Terillon was present.

Passing on to the consideration of purulent effusions, Dr. Ewald begins by reaffirming his opinion that these cases, which seems to have become purulent in consequence of tapping, really contained within themselves the elements of purulency. He especially cautions us to shake our patient before making the trial puncture, lest, owing to position, the solid elements of the pus may have gravitated to the lowest part of the cavity, leaving only a serous supernatant fluid, which would infallibly mislead us if we trusted to it. He considers it all important to find out as early as possible the real nature of the exudation, and counsels the use of a hypodermic or similar syringe for exploration in all cases. "We have punctured," says he, "in hundreds of cases, and have gone into both liver and lung—sometimes on purpose, sometimes accidentally—and have never once seen the slightest harm result therefrom." Great stress is laid on the necessity for absolute cleanliness and the desirability of soaking one's needle in carbolic acid solution just before using it. As to the period at which pus is formed, he gives one case where it was found on the fifth day of the illness; another on the seventh day. Then, as to treatment, some interesting statistics are given, showing that out of forty six cases twenty-six were freely opened, with 12 deaths (46.15 per cent.); nine were aspirated, with seven deaths (77.77 per cent.); and eleven were treated therapeutically, with seven deaths (63.63 per cent.); The first two included the *empyemata necessitatis* (spontaneously opened empyemata), which were naturally not included in the third series. Or, from another point of view, excluding the *empyemata necessitatis*, he gives twenty-one cases freely, opened, with ten deaths (47.74 per cent.); eight cases aspirated with six deaths (75.00 per cent.) ; and seventeen cases (including the *empyemata necessitatis*) treated medicinally, with ten deaths (58.82 per cent.). These figures speak for themselves, and it will be at once inferred that the author strongly advocates a free incision so soon as the diagnosis of pus is established.

Concerning the duration of the disease, he points out the cases which were operated on had an average duration of six to nine months, as against five to six of the non-operated ones; and that the mortality increases in ratio direct with the length of time which is allowed to elapse between the commencement of the disease and the surgical treatment; also, that in young patients recovery is more frequent and more complete than in older ones.

Aspiration of the chest is not considered satisfactory, because, under ordinary circumstances, it is not possible to get rid of all the pus collected unless air be admitted ; and partial evacuation is more or less useless, for the same reason that the partial evacuation of an external abscess would be considered insufficient, The author, as a result of his own observations, then, considers that a free opening into the chest, as soon as the diagnosis is definitely made, offers the best chance for the patient's recovery.

A free exit for the pus may be secured ; and he advises that, if other means fail, Roser's method of resecting a portion of rib should be adopted. The chest-cavity at first is to be washed out twice a day, and afterwards once a day, with such solutions (carbolic acid, Condy's fluid, iodine, iodide of potassium, etc.) as circumstances may suggest, and such constitutional remedies as may seem indicated are to be prescribed.

From what has been said, therefore, it will be seen that there is a wide difference in the results of treatment in these two diseases, and it is to be regretted that they are not alike favorable. Perhaps some will be disappointed in the conclusions which have been drawn concerning purulent effusion into the chest, as to its mortality under any method of treatment, but especially when treated by the method of aspiration. Still it is a fact that only about 7 per cent. recover, and some few of these even are doubtful as to their final condition. The cases in which the chest is freely opened do best, and even then only about one-half recover.—*Med. Times and Gaz.*, April 1, 1876.

